# Superficial extraconal blockade for vitreoretinal surgery

**DOI:** 10.4103/1658-354X.71346

**Published:** 2010

**Authors:** W. Riad, E. Abboud1, E. Al-Harthi, E. Kahtani, N. Ahmed

**Affiliations:** *Department of Anesthesiology King Khaled Eye Specialist Hospital, Riyadh, Kingdom of Saudi Arabia*; 1 *Department of Ophthalmology, King Khaled Eye Specialist Hospital, Riyadh, Kingdom of Saudi Arabia*

**Keywords:** *Peribulbar blockade*, *retinal surgery*, *scleral buckle*, *short needle*

## Abstract

**Context:**

Needle length plays an important role for the success of ophthalmic block. The standard practice is to use 25 mm needles length; however, unnecessarily long needles may increase the risk of complications especially in the presence of staphyloma or previous scleral buckle.

**Aims:**

This work was designed to compare the efficacy of using 15 and 25 mm needle in performing extraconal block for patients undergoing vitreoretinal surgery.

**Settings and Design:**

Prospective randomized double blinded study.

**Materials and Methods:**

A total of 120 patients were enrolled in this study and were divided in two groups. In group (1) extraconal block was performed using 25 mm needle, while in group (2) 15 mm needle was used. After primary injection, assessment of the block was done by an anesthesiologist who was unaware of the needle used. If satisfactory akinesia was not achieved a supplementation was provided. At the end of the procedures, patients and surgeons were asked to assess their pain and satisfaction with the anesthetic technique.

**Statistical Analysis used:**

The sample size calculation using N-Quary version 4. Numerical and categorical data were analyzed using an independent sample, a two-tailed *t*-test, and chi-square test, respectively.

**Results:**

The volume of primary injectable was significantly higher in group 2. The two groups were comparable as regards total volume of local anesthetic, supplementation rate, akinesia, pain score, and surgeon satisfaction.

**Conclusions:**

Using 15 mm needle length to perform extraconal blockade for posterior segment procedures is equally effective to 25 mm needle.

## INTRODUCTION

A majority of patients presenting for retinal procedures are either elderly or having several co-morbidities in which general anesthesia carries a higher risk. A peribulbar technique was successfully used for this type of patients.[1] Posterior segment surgeries are associated with extensive ocular manipulations, generate more pain and are lengthy compared to phacoemulsification procedures. Therefore an adequate intraoperative anesthesia technique is highly desirable.

Although peribulbar block is considered as an effective technique, the blind administration of long needle into the orbital cavity has been associated with sight-threatening complications including inadvertent globe penetration, optic nerve injury, brainstem anesthesia, retrobulbar hemorrhage, and retinal vascular occlusion. On the other hand, life-threatening complication including seizures and cardiopulmonary arrest has been reported.[2] It had been observed that there are variations in the technique of performing peribulbar blocks among different centers in term of selection of site of injection, concentration and volume of local anesthetic solution, type, and length of needle.

Needle length is important for the safe conduct of peribulbar blocks. Theoretically, shorter needles may reduce damage to vital orbital structures and decrease complications. Previously, some authors recommended that maximum penetration of needle from orbital rim should not be greater than 31 mm.[3] At present, the standard practice is to use 25 mm needle during administration of extraconal injection but complications continued to be reported.[4] The aim of this work was to compare the efficacy of using 15 to 25 mm needle in performing extraconal block for patients undergoing vitreoretinal surgery.

## MATERIALS AND METHODS

After approval of the hospital Research and Human ethics committees as well as informed patient consent, 120 patients undergoing pars plana vitrectomy under regional anesthesia were enrolled in this randomized double-blinded prospective study. The patients were randomly divided into two groups using a sealed envelope technique (60 each). In group 1, peribulbar block was performed using standard sharp disposable 25 mm, 25 G needle while in group 2, 15 mm, 25 G needle was used. Patients allergic to local anesthetic solutions, with local sepsis, serious impairment of coagulation, and orbital abnormalities, or who were unable to cooperate in maintaining a relatively motionless supine position or who refused the anesthetic technique were excluded from the study.

The patients were requested to be fasting for 6 h preoperatively and were premedicated with hydroxyzine, paracetamol and, codeine (Revacod) 1 h before surgery according to the standard policy of the hospital. On arrival at OR holding area, standard monitoring of vital signs were commenced and an intravenous cannula was placed. Initial globe movements in all directions of gaze (superior, inferior, medial, and lateral) were assessed.

Our technique of peribulbar block involves insertion of the needle through the lower eyelid as far lateral as possible in the inferotemporal quadrant. After negative aspiration, a volume of 8-10 ml of local anesthetic solution (Bupivacaine 0.5%, Lidocaine 2% 3:2 with hyaluronidase 5 unit/ml) was injected. Digital pressure was applied by the thumb and index fingers around the needle hub during injection. Ocular movements were assessed at 5 and 10 min after the block. A simple akinesia score was used for assessment of the block.[5] Eye movement in four directions is assessed-inferior, superior, medial, and lateral. Normal movement is scored at 2 and reduced movement at 1 and flickering movement and/or akinesia is scored at zero. Internationally using this scale, an ocular mobility score of ≤3 is acceptable as indicative of successful block. For the purpose of surgery, a score of 1 or 0 out of 8 was acceptable. Determining the score was done by one of the investigator anesthesiologists who was unaware of the length of the needle used for performing the block. If after 10 min. the block is inadequate for surgery, supplementary anesthesia was provided by addition of more anesthetic solution (5-10 ml) either medial or superior-medial to the globe using the same needle used for the primary injection.

Anesthesia-related scores of satisfaction was determined postoperatively by surgeons using horizontal visual scale from 0 (total dissatisfaction) to 10 (total satisfaction).[6] For better analysis of surgeon satisfaction, the scale reclassified into three point: excellent (score 8-10), good (6-8), and poor (0-5). Any complication and additional supplements of local anesthetics which was provided by the surgeon intraoperatively were documented. At the end of the procedure, patients were asked by the surgeon to rate their degree of intraoperative pain using 10 points Verbal analogue scale from 0 (no pain) to 10 (the most intense pain imaginable).[7]

The results were analyzed using the Statistical Package for Social Science for Windows version12 (SPSS Inc., Chicago, IL, USA). The sample size calculation using N-Quary software version 4 indicated that 120 patients were required to detect a 0.5 difference in the mean of the simple akinesia score between the two groups, when alpha error =0.05, and type II error 0.1. Numerical data were expressed as a mean and standard deviation (SD) and were analyzed using an independent sample, two-tailed *t*-test. On the other hand, categorical data were expressed as number and percentage. Ordinal and nominal data were compared using the chi-square test. A *P* value of 0.05 was used as the level of significance.

## RESULTS

A total of 120 patients were enrolled in this study. Demographic and clinical data are shown in Table 1. The two test groups were comparable for age, weight, height, sex, American Society of Anesthesiologist (ASA) physical status grade, and duration of anesthesia.

**Table 1:** Demographic and clinical data

	Group 1 (25 mm) (n=60)	Group 2 (15 mm) (n=60)	*P* value
Age (years)	59.7 (9.6)	61.7 (9.5)	0.25
Weight (kg)	74.4 (13.6)	77.1 (19.8)	0.39
Height (cm)	160.8 (9.4)	158.8(16.2)	0.41
Sex			0.18
Male	35 (58.3%)	43 (71.7%)	
Female	25 (41.7%)	17 (28.3%)	
ASA grade			0.34
1	2 (3.3%)	3(5%)	
2	17 (28.3%)	16 (26.7%)	
3	41 (68.3%)	38 (63.3%)	
4	0	3(5%)	
Duration of anesthesia (min)	116.3 (30.4)	108.1(33.8)	0.15

Data expressed as mean (SD) or number (percentage)

The volume of local anesthesia injected, supplementation rate, and akinesia score are shown in Table 2. The initial volume of local anesthetic was significantly higher in the 15 mm needle length group (*P* value=0.035). However, no difference detected as regard the total volume of local anesthetic used (*P* value=0.70). The number of patients who required supplementation and total number of supplements were comparable between the two groups (*P* value=0.51 and 0.63, respectively). No significant difference was observed between the groups as regard akinesia score after 5 and 10 min following injection (*P* value=0.74 and 0.93, respectively).

**Table 2:** Volume of local anesthesia injected, supplementation rate, and akinesia

	Group 1 (25 mm) (n=60)	Group 2 (15 mm) (n=60)	*P* value
Initial volume injected Infero-lateral approach (ml)	9.18 (1.0)	9.57 (0.85)	0.035
Total volume injected (ml)	10.8(3.9)	11.0 (3.7)	0.70
No. of patients required supplementary injection	15 (25%)	11(18.3%)	0.51
Total no. of supplements	17	12	0.63
Akinesia after 5 min	0.88 (1.4)	0.80 (1.2)	0.74
Akinesia after 10 min	0.60 (1.1)	0.62 (1.1)	o.93

Data expressed as mean (SD) or number (percentage)

Intraoperative data are shown in Table 3. There was no difference between the groups as regard patient’s intraoperative pain and surgeon satisfaction grade (*P* value=0.21 and 1.00, respectively). The groups were comparable as regards intraoperative supplementation rate, time after initial block, and volume of the supplement (*P* value=0.43 and 0.55 and 0.36, respectively).

**Table 3:** Intraoperative events

	Group 1 (25 mm) (n=60)	Group 2 (15 mm) (n=60)	*P* value
Pain score	0.7 (1.89)	0.3 (1.29)	0.21
Surgeon satisfaction grade			
Excellent	58 (96.7%)	57 (95%)	1.00
Good	2 (3.3%)	3(5%)	
Poor	0(0%)	0(0%)	
Intraoperative supplement by the surgeon	5 (8.3%)	2 (3.3%)	0.43
Time of intraoperative supplement after the initial block (min)	92.8(23.3)	81.5(12.0)	0.55
Volume of intraoperative supplement (ml)	3.40 (1.6)	2.5(0.7)	0.36

Data expressed as mean (SD) or number (percentage)

No major life-threatening or block-related complications were noted.

## DISCUSSION

The results of the current study showed that the use of a 15 mm needle for posterior segment procedures gives a comparable result to a 25 mm needle in terms of volume injected of local anesthetic, akinesia, supplementation rate, intraoperative pain, and surgeon/patient satisfaction. Gills *et al*,[8] divide the orbit into three spaces (anterior, mid, and posterior) for better appreciation of the relationship of injection site. The anterior orbit ends 2-5 mm anterior to the equator of the globe and is filled primarily with connective tissue while the mid-orbit ends posteriorly about 10-12 mm behind the posterior surface of the globe. It contains primarily muscle bellies and adipo-connective tissue. Insertion of longer needles deep into the orbit increases the potential of injury to important structures and that limitation of the depth of needle insertion may limit needle injury.[9] Hamilton demonstrated that a needle longer than 31 mm increased the risk of direct injection into the subarachnoid space and injury to the optic nerve.[10] Ben- David reported that ophthalmic blocks are associated with most of disabling injuries of regional anesthesia in ASA Closed Claims.[11] Scott and colleagues[12] demonstrated that a 16 mm needle reaches to the vicinity of the orbital equator and cannot pass beyond it, while the longer needle of 25 mm maybe advanced beyond this area into the more dangerous retrobulbar space and may pass into the inferior orbital floor reducing the spread of local anesthetic around the globe itself.

The findings of this work are consistent with Rizzo *et al*,[13] who demonstrated 78.6% of patients had a motor block of more than 80% after 5 min and after 7 min 100% of patients had adequate anesthesia to proceed with and complete the surgery using 16 mm needle with infero-medial approach for anterior segment surgery. Scott and associates[12] demonstrated effective results with a 16 mm needle and attributed their success to effective anatomic placement within the orbit allowing access to the retrobulbar space via fascial septae. It had been clearly demonstrated that fascial sheath around the eyeball extends to the recti and orbiculars muscles which guides the local anesthetic preferentially to those muscles producing akinesia. On the other hand, Van den berg audit from our institution showed that superficial injection with 15mm needle produced significantly lower degree of akinesia and required a higher supplementation rate (64%) as compare to supplementation rate with deeper injection with 25 mm needle (44%).[14] The same mixture of local anesthetic was used for both studies. The difference could be attributed to application of digital compression with the thumb and index fingers around the needle hub at the time of injection, which promoted the degree of akinesia by spreading the solution deeper and posterior to the equator of the globe through the gaps in the orbital septum rather than allowing it to accumulate in the anterior orbit. This simple modification allows larger number of local anesthetic molecules to be distributed around the eyeball in order to improve the success rate of peribulbar block.

The observation in this study that relatively larger volumes of local anesthetic (approximately 11 ml) are used after supplementation compared to lower volume (5-6.5 ml) used by Rizzo *et al*,[13] could be attributed to population differences, point of injection, different akinesia scoring systems used, and type of the procedure. In the same vein, Knight and his colleague used a total volume between 7 and 10 Ml for vitreoretinal surgery with 25 g 1-inch needle.[15] On the other hand, Calenda and colleagues[16] used a mean volume of 17 Ml by the double injection technique (inferior and superior injections) with 35 mm needle to achieve adequate anesthesia in 85% of their patients. The result of this study and similar studies[12,13,15,16] showed the efficacy of the technique. Hence, larger studies are needed to prove the safety. Therefore, these facts could be considered as an argument for replacing longer needles (25 mm) with a shorter needle (15 mm) in current practice.

## CONCLUSION

Peribulbar anesthesia with 15 mm needle, using the combination of 2% lidocaine, 0.5% bupivacaine, and 5 unit/ml hyaluronidase, provides excellent surgical conditions, akinesia, and analgesia in patients undergoing posterior segment surgery and it is equally effective to 25 mm needle.
